# Deep Learning-Based Contact Force Control for a Robotic Leg

**DOI:** 10.3390/s26051473

**Published:** 2026-02-26

**Authors:** Hyoseok Lee, Dongmin Baek, Hyeokjun Kwon, Hyun-min Joe

**Affiliations:** 1 Department of Robot and Smart System Engineering, Kyungpook National University, Daegu 41566, Republic of Korea; erlangen416@gmail.com (H.L.); hjkwon.knu@gmail.com (H.K.); 2 Autonomous Manufacturing System Section, Medium-Size Ship Automation Innovation Department, HD Hyundai Heavy Industries, Ulsan 44113, Republic of Korea; dongminb@hd.com

**Keywords:** admittance, deep learning, force control, robot control, robot learning

## Abstract

This paper proposes a learning-based contact force controller using deep neural networks (DNN) and a PI controller. Stable contact force control between the foot and the ground is essential for humanoid robots to maintain balance during bipedal walking. While admittance controllers have been extensively employed for contact force control in humanoid robots, their performance is limited by the high nonlinearity inherent in robot systems. To overcome these limitations, we propose a deep neural network (DNN)–based inverse model, which leverages input–output data that inherently capture system nonlinearities. The proposed learning-based contact force controller computes the target foot height based on the target force, measured force, and measured foot height, without relying on a dynamic model of the articulated robotic leg. Furthermore, a PI controller is integrated to mitigate steady-state errors. Experimental comparisons between the proposed controller and an admittance controller were conducted using an articulated robotic leg. Compared with an admittance controller, the proposed method reduced overshoot by 96% and settling time by 61% on average in step responses and decreased force-tracking RMSE by 66.3% on average across both step and sinusoidal experiments.

## 1. Introduction

Stable control of the contact force between the foot and the ground is essential for humanoid robots to maintain balance and achieve robust bipedal locomotion. To address this challenge, previous studies have focused on mechanical design and control algorithms. Mechanical design–based methods typically incorporate elastic materials or structures into robot hardware to absorb shocks during walking. The Honda P2 humanoid robot has employed a rubber material as a mechanical filter in the foot to absorb shocks from contact with the ground [1]. A study emulated the arch structure of the human foot, incorporating springs to absorb impacts on the humanoid robot’s foot from the ground [2]. Series elastic actuators mitigated shocks through elastic materials placed serially between motors and robot links [3]. Buchner et al. presented an electrohydraulic musculoskeletal robotic leg with tunable stiffness and capacitive self-sensing that achieved agile, energy-efficient hopping over diverse terrains using a single open-loop force-control command set [4]. However, these approaches are highly specialized and hardware-intensive, which can limit their generalizability. Moreover, modifying the mechanical design alone does not guarantee precise control over the ground reaction force and can even increase system nonlinearity due to the complexity and elasticity of the robot structure.

Compliance control algorithms have been widely adopted to regulate contact forces. The BHR-6P humanoid robot has utilized a viscoelastic model for ankle compliance control [5]. A typical example of compliance control is admittance control, which adjusts the system’s position and speed in response to external disturbances [6]. Admittance controllers have been utilized in compliance control for the feet of robots such as KAIST’s DRC HUBO [7] and the HRP-4 by AIST and Kawada [8]. However, challenges in parameter selection for highly nonlinear systems compromise the performance of this technique.

Model predictive control (MPC) has also been widely studied for contact force control of legged robots because it can explicitly handle dynamic and contact-related constraints in an optimization framework [9]. Romualdi et al. proposed an online nonlinear centroidal MPC with step adjustment for humanoid locomotion, enabling real-time adaptation of motion and foothold planning under changing contact conditions [10]. Sombolestan and Nguyen presented an adaptive force-based control method for dynamic legged locomotion over uneven terrain, showing robust contact-force regulation against terrain-induced disturbances [11]. Since MPC requires solving an optimization problem at each control cycle, the achievable control rate is limited by computation and latency, and performance is sensitive to model mismatch.

To overcome the limitations of traditional controllers in nonlinear systems, learning-based approaches such as reinforcement learning and deep neural networks have been widely investigated. Reinforcement learning optimizes a control policy through trial-and-error interactions with the environment to maximize a defined reward function. Portela et al. proposed RL for direct force control in legged manipulation and demonstrated compliant whole-body behavior on a quadruped manipulator platform [12], while Zhang et al. introduced FALCON, a dual-agent RL framework for force-adaptive humanoid loco-manipulation under 3D end-effector force interaction and external force disturbances [13]. However, reinforcement learning requires complex reward shaping or sophisticated sim-to-real transfer techniques to ensure stability.

Therefore, deep neural network (DNN)–based control methods have been utilized because they can effectively learn system dynamics from collected data [14]. Data collection for neural network training can be performed using online or offline methods. The online method continuously updates the model’s weights in real time. Some studies have explored the online training of DNN models to dynamically adjust admittance control coefficients [15], as well as lifelong deep learning to enhance the trajectory-tracking performance of manipulators [16]. Phan et al. proposed a disturbance-observer-based adaptive neural backstepping integral sliding-mode controller for a flexible-joint robot with hydraulic actuator dynamics, in which neural-network parameters are updated in real time to compensate for model uncertainties and external disturbances [17]. However, since this research integrates multiple control components, it introduces many tuning parameters, increasing implementation complexity. Furthermore, online methods risk converging to local minima, potentially impacting the model’s generalizability. The offline method computes and updates weights based on the entire collected dataset, ensuring broader generalization and, thus, versatility for various systems.

DNN-based control can be classified into indirect and direct methods. Indirect methods involve using neural networks for system modeling [18], whereas direct methods utilize the neural network itself as a controller. Direct control using DNNs has demonstrated improved force control performance in nonlinear hydraulic actuators [19] and enabled robust force control in nonlinear twisted-string actuators [20]. However, direct control approaches suffer from steady-state errors.

In this study, we propose a deep neural network (DNN)–based inverse model to address nonlinearities arising from contact between an articulated robotic leg and the ground. Furthermore, a PI controller is integrated with the DNN-based inverse model to eliminate steady-state errors, constituting a learning-based contact force controller.

The main contributions of this research are as follows:

1. A DNN-based inverse model is proposed to track the target force via direct methods.
2. A PI controller is incorporated to resolve steady-state errors by compensating for the input values of the DNN-based inverse model.
3. Reference force–tracking experiments are conducted to evaluate the performance of the proposed learning-based contact force controller.

The remainder of this paper is structured as follows: Section 2 describes the design of the robotic leg, the admittance controller, and the learning-based contact force controller. Section 3 details the experiments conducted to compare the performance of different controllers. Section 4 discusses the experimental results. Finally, Section 5 presents the conclusions from the study.

## 2. Methodology

### 2.1. Articulated Robotic Leg Design

**Figure 1:**
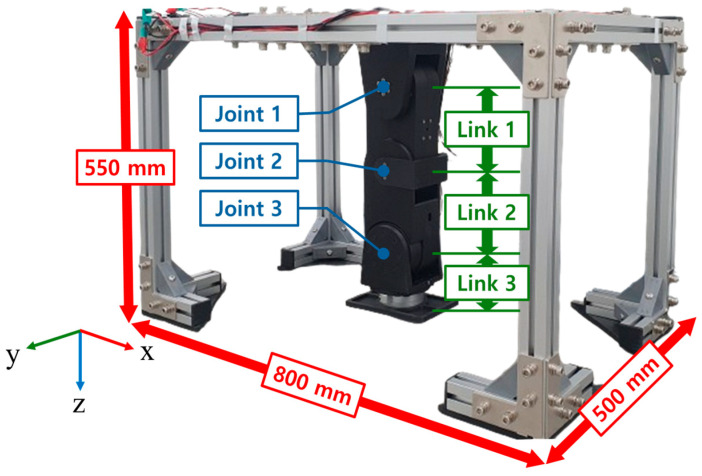
Robotic leg fixed on aluminum frame.

A three-degree-of-freedom (3-DOF) robotic leg was designed and fabricated to serve as the hardware system for the contact force control experiments. Figure 1 illustrates the hardware configuration of the robotic leg fixed on an aluminum-frame testbed. The link lengths are L1 = 0.15 m, L2 = 0.15 m, and L3 = 0.11 m, and the total weight of the robotic leg is 5.25 kg. The leg is equipped with three motors and a single force/torque (F/T) sensor. The links of the robotic leg were 3D-printed using Onyx as the printing material. The frame had a rectangular parallelepiped shape with dimensions of 800 mm × 500 mm × 550 mm.

**Figure 2:**
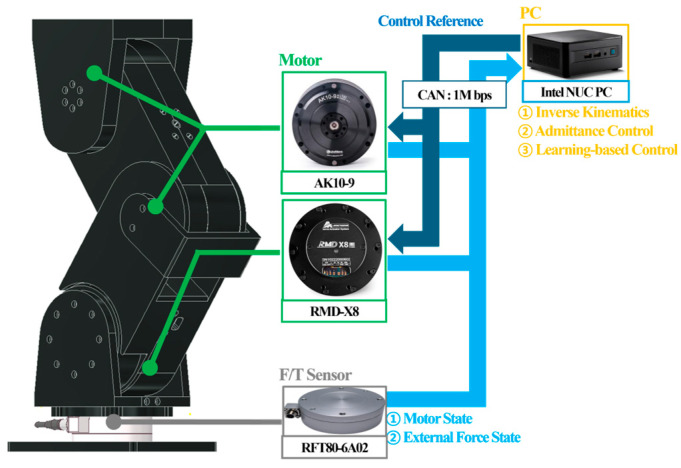
Hardware control system for robotic leg.

Figure 2 illustrates the hardware control system of the robotic leg. The leg used 1 Mbps CAN to communicate with the control PC (Intel; Santa Clara, CA, USA), which handled both the high-level and low-level control tasks. High-level control includes kinematic calculations, admittance control, and learning-based contact force control, whereas low-level control includes motor control and the reception of measurement values from the motor and F/T sensor. Table 1 lists the specifications of the motor and F/T sensor for each joint. For low-level joint control, we utilized the actuators’ built-in position controllers with factory default PID gains, which were maintained constant throughout all experimental conditions.

### 2.2. Admittance Controller

**Figure 3:**
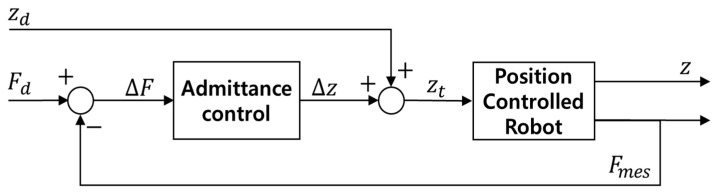
Block diagram of position-controlled robot system equipped with admittance controller.

The equation for the admittance controller is as follows:

(1) $$M\left(∆\ddot{z}\right)+B\left(∆\dot{z}\right)+K\left(∆z\right)=∆F$$

where *M* is the mass coefficient, *B* is the damping coefficient, *K* is the spring coefficient; $∆\ddot{z}$, $∆\dot{z}$, and ∆z represent the changes in acceleration, velocity, and position, respectively, in the z-direction; and ∆F represents the force error. As this study focuses on regulating contact force exclusively along the vertical *z*-axis to ensure stable ground interaction, the admittance controller was intentionally designed as a 1-DOF baseline along the same axis. The admittance controller was tuned to follow a step response of 50 N when the robotic leg encountered an obstacle 0.06 m high. The coefficients of the admittance controller were *M* = 2, *B* = 200, and *K* = 80. Figure 3 presents a block diagram of the robotic leg equipped with the admittance controller, where $F_{d}$, $F_{mes}$, and ∆F represent the target force, measured force, and force error, respectively, while ∆z, $z_{d}$, $z_{t}$, and z denote the change in height, the contact height corresponding to the robotic leg and the obstacle, the input height, and the measured height, respectively. ∆F constitutes the input to the admittance controller, while ∆z is its output; ∆z is added to $z_{d}$ to calculate $z_{t}$, which serves as the basis for the robot’s position control.

### 2.3. First Learning-Based Contact Force Controller

The fundamental objective of force control is to minimize the error $e\left(t\right)=\;F_{d}\left(t\right)-\;F_{mes}(t)$, where $F_{d}\left(t\right)$ is the target force and $F_{mes}(t)$ is the measured force. In conventional approaches, such as admittance control, the control input u(t) is typically generated based on this error through a feedback law:

(2) u(t)=G(e(t))

where G represents the controller transfer function. In contrast, the proposed method is based on a data-driven inverse model. It directly computes the required target position $z_{d}$ from the target force $F_{d}$, measured force $F_{mes}$, and measured height z, effectively functioning as a feedforward controller. To implement this learning-based controller, the development process involved acquiring the system dynamics through data collection and subsequent training.

**Figure 4:**
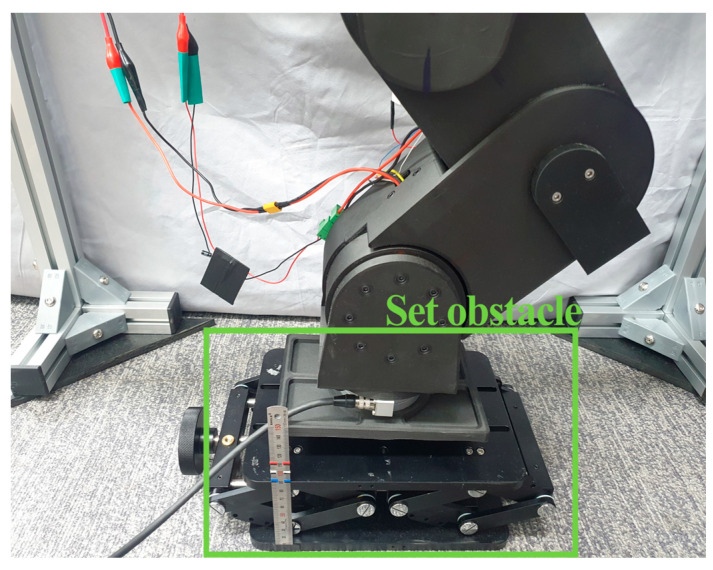
Height-adjustable obstacle installed below robotic leg.

**Figure 5:**
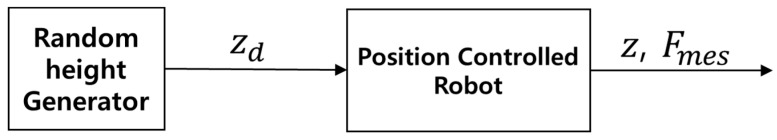
Data collection process for first DNN-based inverse model.

The proposed controller’s operation involved data collection and training in two stages. In the first stage, the data to train the first DNN-based inverse model were collected. As shown in Figure 4, a height-adjustable obstacle was positioned beneath the retracted robotic leg; the obstacle’s height was set to 0.06 m. The end effector of the robotic leg was lowered at 0.0215 m/s. Upon contact with the obstacle, indicated by the F/T sensor’s z-directional measurement exceeding 30 N, the robotic leg’s target height was randomly selected through linear interpolation, according to the following equation:

(3) $$z_{input}=z_{0}+\frac{\left(z_{R}-z_{0}\right)\left(T_{input}-T_{0}\right)}{T_{R}-T_{0}}$$

where $z_{input}$ is the height set for the robotic leg, $T_{input}$ is the current time, $z_{0}$ is the height in the initial state, $T_{0}$ is the time in the initial state, $z_{R}$ is a random height, and $T_{R}$ is a random time. When $z_{R}$ and $T_{R}$ were randomly selected within the defined range, $z_{input}$ was applied to the robotic leg for a duration of $T_{R}$. When the time exceeded $T_{R}$, $z_{R}$ and $T_{R}$ were updated to new random values, and the previous values were used as $z_{0}$ and $T_{0}$ for the next interpolation. The range of $T_{R}$ was 0.5–2.5 s, while that of $z_{R}$ was 0 to −0.009 m relative to the contact height corresponding to the robotic leg and the obstacle. To ensure maximal diversity in the selection of $z_{R}$ within the defined range, the next $z_{R}$ value was constrained to differ from the previous value by at least 0.004 m upon each update. Figure 5 illustrates the process where the random height generator provides $z_{d}$ directly into the robot to output $z,\;F_{mes}\;$ data for the training of first the DNN-based inverse model.

**Figure 6:**
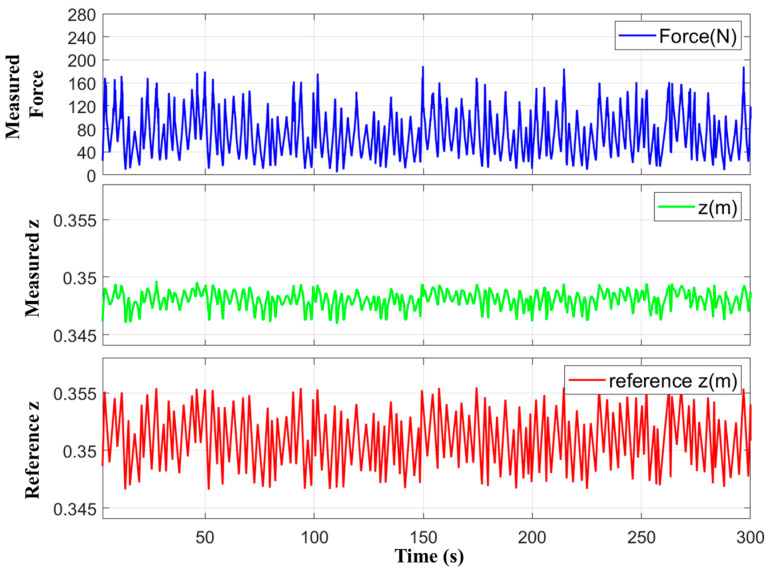
Collected training data.

Data were collected at a frequency of 500 Hz for a total duration of 5 min, which yielded a dataset comprising 150,347 data points. The collected data included the target height input to the robotic leg, the measured force, and the measured height. A low-pass filter with a 10 Hz cutoff frequency was applied to the sensor measurements to mitigate noise. Figure 6 presents graphs of the collected data. The equations of the first DNN-based inverse model are defined:

(4) $$X={\left[F_{d}(t+10),\ldots ,F_{d}\left(t+1\right),F_{mes}\left(t\right),z(t)\right]}^{T}$$

(5) $$T_{l}\left(X\right)=W^{\left(l\right)}X+b^{\left(l\right)}$$

(6) $$\rho \left(x\right)=\max\left(0,x\right)$$

(7) $$z_{d}(t)=E\left(X\right)=T_{L}\rho \left(T_{L-1}\rho \left(\cdots \rho \left(T_{1}(X)\right)\right)\right)$$

where X is the input vector, $F_{d}\left(t+10\right),\ldots ,F_{d}\left(t+1\right)\;$ is the future target force trajectories, $F_{mes}(t)$ is the measured force, and z(t) is the measured height. The function $T_{l}$ represents the affine-linear functions, $W^{\left(l\right)}$ are the weight matrices, $b^{\left(l\right)}$ are the bias vectors, ρ is the activation function, L is the number of layers, l=1,⋯,L, and $z_{d}$(t) is the target height. The neural network is represented by the function E. The number of steps for the future target force trajectory was set to 10, following an existing approach for similar systems [21].

**Figure 7:**
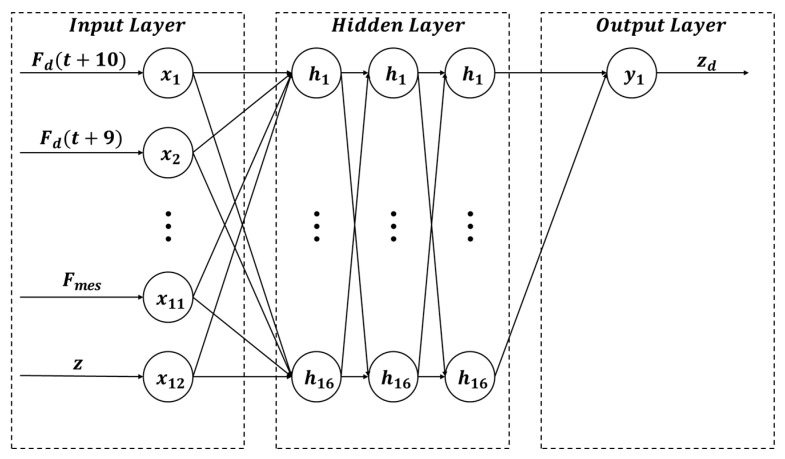
Architecture of first DNN-based inverse model.

**Figure 8:**
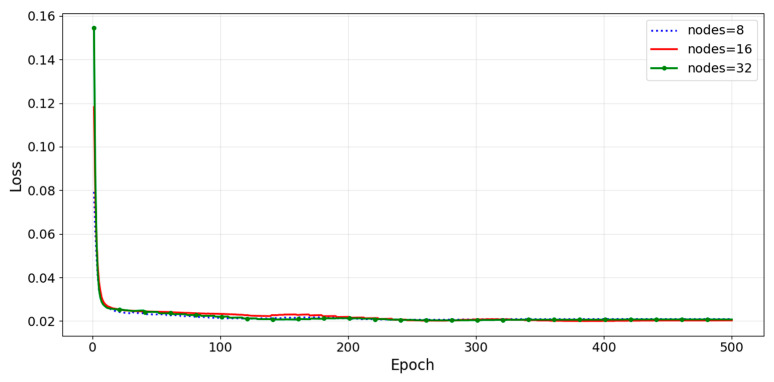
Comparison of the validation loss for different numbers of neurons per hidden layer.

Figure 7 illustrates the architecture of the first DNN-based inverse model, which consists of an input layer, three hidden layers, and an output layer. Following prior studies on nonlinear inverse modeling [19,22], the network depth was fixed to three hidden layers. To determine the hidden-layer width, we evaluated 8, 16, and 32 neurons per layer. The minimum validation losses were $2.06\times 10^{-2},2.00\times 10^{-2},$ and $2.03\;\times \;10^{-2}$, respectively, as illustrated in Figure 8. Therefore, 16 neurons were applied across all layers [23]. The Rectified Linear Unit (ReLU) was employed as the activation function to ensure training stability [24]. The collected data were normalized using the z-score method before being input into the network.

The other hyperparameters are shown in Table 2. L1 and L2 regularization were employed to regulate the weights for each layer. The learning rate was multiplied by 0.90 if the validation loss did not improve for 10 epochs. Moreover, early stopping was implemented to prevent overfitting if the validation loss did not improve by more than 0.0001.

During optimization, a gradient clipping of 0.1 was used to prevent gradient explosion. The batch size during training was 128. Furthermore, we adopted the Huber loss function [25], which uses mean squared error (MSE) for small errors and mean absolute error (MAE) for large errors. Equation (8) defines the Huber loss function:

(8) $$L_{d}\left(a\right)=\left\{\begin{array}{c} \;\;\;\;\frac{1}{2}a^{2},\;\;\;\;\;\;\;\;for\;\left|a\right|\leq d\\ \;\;\;\;d\cdot \left(\left|a\right|-\frac{1}{2}d\right),\;\;\;\;\;\;\;\;otherwise \end{array}\right.$$

where a represents the difference between the predicted and actual values, and d is the threshold value. The Huber loss function is sensitive to small errors while remaining robust against outliers. Thus, it increases the stability of neural network training and minimizes the loss value during the training process. The total number of parameters in the proposed neural network was 769. The validation loss was measured to be 0.02 after training.

**Figure 9:**
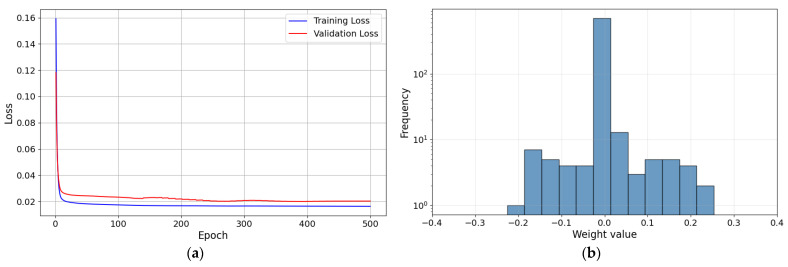
Result of training first DNN-based inverse model; (**a**) Training and Validation loss curves (**b**) Weight distribution plot.

**Figure 10:**
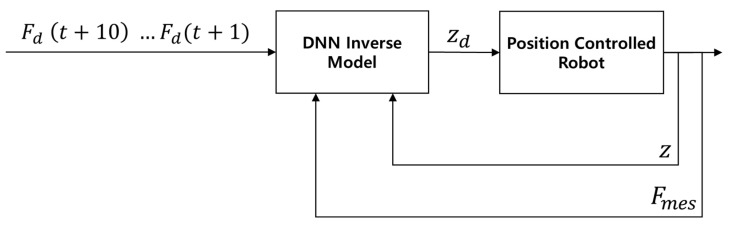
Block diagram of first learning-based contact force controller.

As shown in Figure 9, both training and validation losses decrease rapidly in the early stage and then converge to stable plateaus without divergence. In addition, the learned weight distribution is concentrated around zero with limited tails, and no evidence of weight explosion is observed. This supports the stability of the optimization process. Figure 10 illustrates the structure of the first learning-based contact force controller, which integrates a DNN-based inverse model. $F_{mes}$ represents the measured force; $F_{d}\left(t+10\right)\ldots F_{d}(t+1)$ represent the target force trajectory; and $z_{d}$ and z are the target height and measured height, respectively. The target forces were arranged sequentially in time, yielding a 10-step target-force trajectory. The target force trajectory, measured force, and measured height were then input to the DNN-based inverse model to produce the target height, which is used to control the robotic leg.

### 2.4. Second Learning-Based Contact Force Controller

During the collection of training data for the first learning-based contact force controller, the target height was randomly controlled while the robotic leg remained in contact with the obstacle, and the corresponding force was measured. The intended measured force was determined by experimentally setting the height range for the robotic leg. Adjusting the height of the contacted obstacle during training could alter the range of the intended measured force. To avoid this, the model was trained considering a fixed obstacle height. Therefore, the first learning-based contact force controller exhibited considerable steady-state errors when the obstacle height differed from the value during training. To address this problem, additional data were collected with the first learning-based contact force controller applied, and a new inverse model was trained.

**Figure 11:**
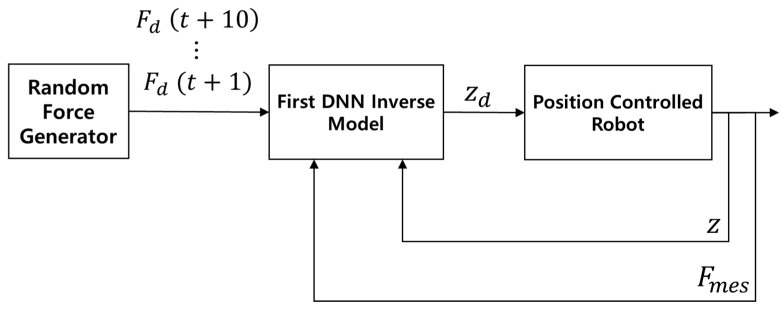
Data collection process for second DNN-based inverse model.

**Figure 12:**
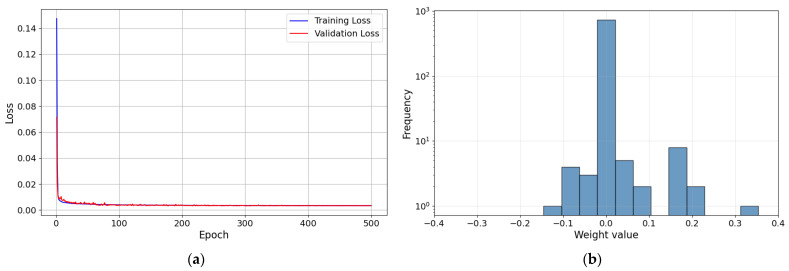
Result of training second DNN-based inverse model; (**a**) Training and Validation loss curves (**b**) Weight distribution plot.

With the first learning-based contact force controller active, random target forces were input through linear interpolation. Equation (9) represents the linear interpolation for the target force:

(9) $$F_{input}=F_{0}+\frac{\left(F_{R}-F_{0}\right)\left(T_{input}-T_{0}\right)}{T_{R}-T_{0}}$$

where $F_{input}$ is the current target force applied to the robotic leg, $T_{input}$ is the current time, $F_{0}$ is the target force in the initial state, $T_{0}$ is the time in the initial state, $F_{R}$ is a random target force, and $T_{R}$ is a random time. The ranges of $T_{R}$ and $F_{R}$ were set to 1–5 s and 15–120 N, respectively. The next $F_{R}$ value was constrained to differ from the previous value by at least 70 N. During data collection, the obstacle height was manually raised by 1 mm/min for 10 min and then lowered by 5 min. During this process, the obstacle height ranged from 0.06 m to 0.07 m. Figure 11 illustrates the process where a random force generator provides $F_{d}(t+10)$,…, $F_{d}\left(t+1\right)$. These are processed by the first DNN-based inverse model to generate $z_{d}\;$ which drives the robot to yield z  and $F_{mes}$, thereby completing the data collection set ($z_{d},\;z,\;F_{mes}$) for the training of the second DNN-based inverse model. The input/output configuration and hyperparameters of the second DNN-based inverse model were identical to those of the first model. The block diagram of the second learning-based contact force controller is the same as that in Figure 10. As illustrated in Figure 12, the second DNN-based inverse model also exhibited stable learning behavior, with smoothly converging training/validation losses and no sign of weight explosion.

## 3. Experiments

### 3.1. Experimental Scenarios

To evaluate the performance of the proposed controller, comparative experiments were conducted against an admittance controller. The end-effector of the robotic leg contacted an obstacle at a speed of 0.172 m/s. The controller was activated when the F/T sensor of the robotic leg detected a z-directional force exceeding 15 N. Each experiment lasted up to 18 s, whereafter the robotic leg returned to its initial position. There are four distinct experiment scenarios: step-force tracking at 50 N with a 0.06 m obstacle, step-force tracking at 50 N with a 0.07 m obstacle, sinusoidal-force tracking with $F_{d}\left(t\right)=15\sin\left(\pi t\right)+65\;(0.5\;Hz)$ with a 0.06 m obstacle, and sinusoidal-force tracking with $F_{d}\left(t\right)=15\sin\left(2\pi t\right)+65(1\;Hz)$ with a 0.06 m obstacle. The verification criteria for the step-response experiment were root MSE (RMSE), overshoot, and settling time (within a 5% error). For the sinusoidal tracking experiment, the verification criterion was RMSE.

### 3.2. PI Controller

**Figure 13:**
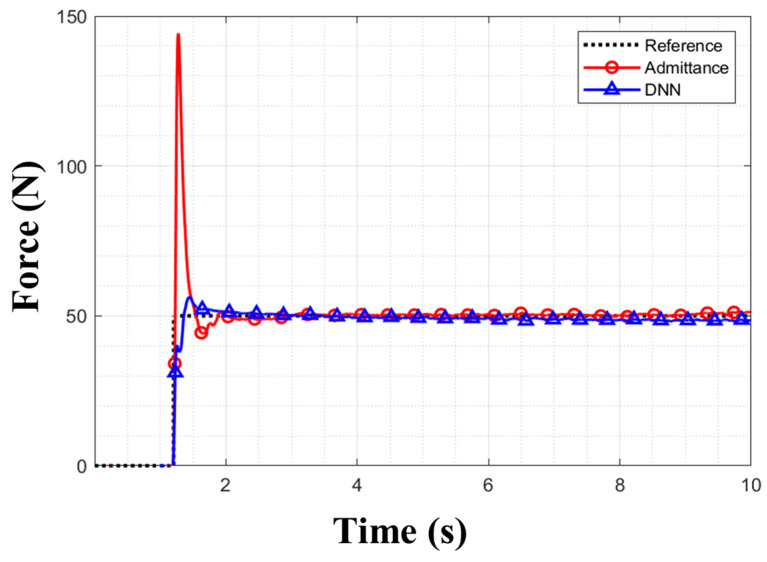
Comparison of force tracking between controllers for 50 N step reference at 0.06 m obstacle height.

Table 3 shows the experimental results obtained when a target force of 50 N was applied at a height of 0.06 m. Figure 13 presents the corresponding force-tracking graph for each controller. While the proposed controller tracks reference force substantially better than admittance, it exhibited characteristics of a feedforward controller, which causes steady-state errors of 2 N. To overcome this, a PI controller was utilized to compensate for the steady-state error. The PI controller was applied for both the first and second learning-based contact force controllers.

**Figure 14:**
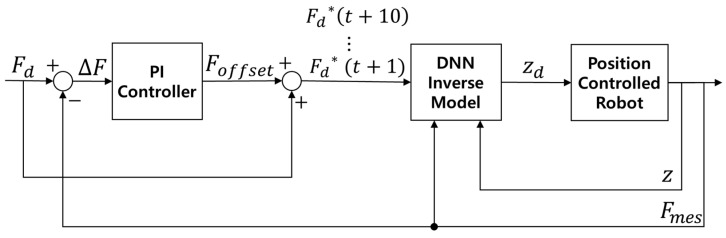
Block diagram of learning-based contact force controller with integrated PI controller.

Figure 14 illustrates the structure of the learning-based contact force controller, which integrates a DNN-based inverse model and PI controller. $F_{d}$, $F_{mes}$, and ∆F represent the target force, measured force, and force error, respectively; $F_{offset}$ denotes the PI controller’s output; ${F_{d}}^{*}\left(t+10\right)$… ${F_{d}}^{*}(t+1)$ represent the compensated target force trajectory; and $z_{d}$ and z are the target height and measured height, respectively. The difference between $F_{d}$ and $F_{mes}$, i.e., ∆F, was input to the PI controller, which generated the output $F_{offset}$. The compensated target force was calculated by summing $F_{d}$ and $F_{offset}$, and a trajectory of 10 compensated target forces was generated. The compensated target force trajectory, measured force, and measured height were then input to the DNN-based inverse model to produce the target height, which is used to control the robotic leg. The PI gains were selected heuristically through step-response tests. Since the primary objective of the PI controller is to eliminate the steady-state error that the DNN-based inverse model cannot track, the I gain was prioritized and set relatively higher than the P gain. The P gain was increased until a fast response was achieved without excessive overshoot or oscillation, after which I gain was raised to remove the steady-state error. The P and I gains of the PI controller were set to 1.1 and 1.7 for the first learning-based contact force controller, respectively, and 0.1 and 1.0 for the second learning-based contact force controller, respectively.

To evaluate the impact of the PI controller on contact force control performance, we conducted a step-response experiment that involved tracking a 50 N target force with and without the PI controller enabled.

**Figure 15:**
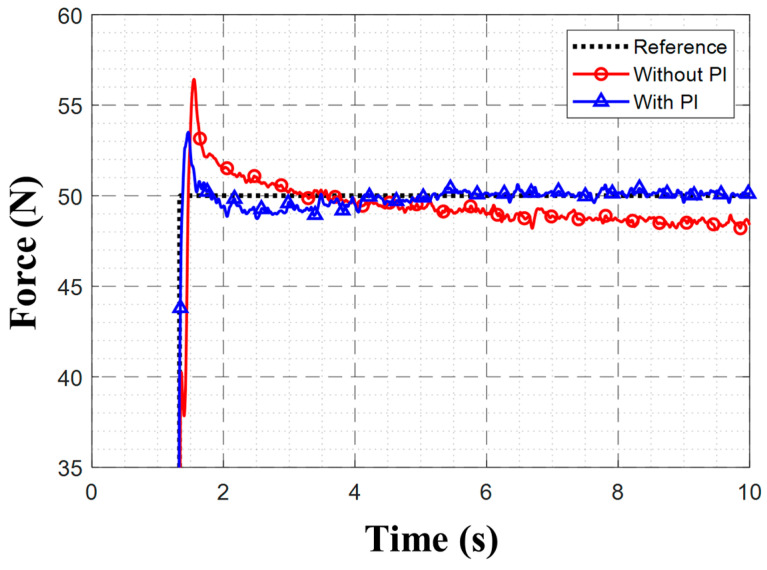
Force-tracking graphs for proposed controller with and without PI controller for 50 N reference at 0.06 m obstacle height.

Table 4 shows the experimental results for a 50 N target force applied at a 0.06 m height, highlighting that the incorporation of the PI controller reduced the RMSE, overshoot, and settling time substantially. Figure 15 shows the force-tracking graphs with and without the PI controller. Integrating the PI controller eliminated the steady-state error, driving the measured force to converge to the 50 N target.

### 3.3. Evaluation of Performance for the Learning-Based Contact Force Controller with PI Controller

**Figure 16:**
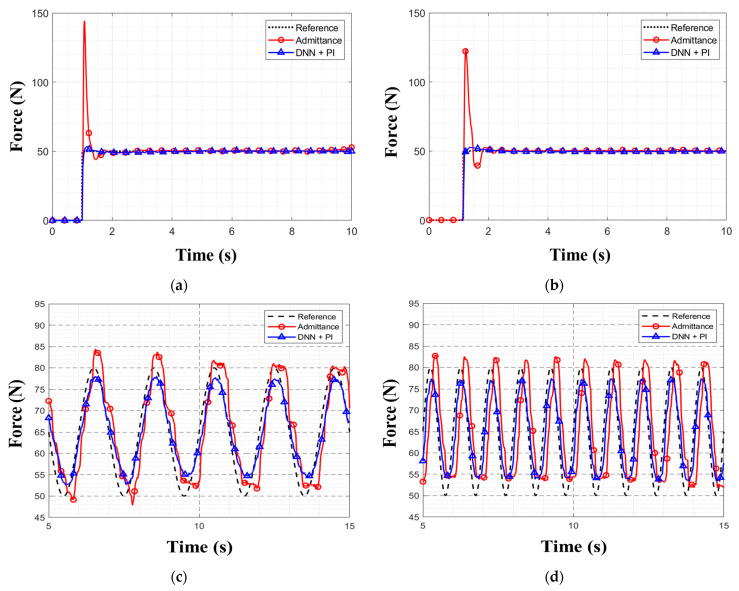
Force-tracking responses of the admittance controller and the proposed controller (DNN + PI): (**a**) 50 N step at a 0.06 m obstacle; (**b**) 50 N step at a 0.07 m obstacle; (**c**) 0.5 Hz sinusoid at 0.06 m obstacle; (**d**) 1 Hz sinusoid at 0.06 m obstacle.

Table 5 summarizes the tracking performance of the admittance controller and the proposed controller (DNN + PI) across step and sinusoidal tests. In Figure 16a,b depict step responses at obstacle heights of 0.06 m and 0.07 m, respectively, while (c) and (d) show 0.5 Hz and 1 Hz tracking, respectively. As evident from the data, the proposed controller achieved a considerably lower RMSE, overshoot, and settling time than the admittance controller in step responses. For the sinusoidal tests at 0.06 m, the proposed controller achieved lower RMSEs than the admittance controller for both 0.5 Hz and 1 Hz.

## 4. Discussion

Based on the results of the step-response experiment comparing the proposed controller with and without the PI controller, the incorporation of the PI controller enhanced performance across all metrics. Whereas a steady-state error of 2 N was observed without the PI controller, the integration of the PI controller eliminated this error, reduced the overshoot, and enabled rapid convergence to the target force. This is because the PI controller generated a compensated force trajectory based on the error between the target force and the measured force, which was input into the DNN-based inverse model.

In the step-response experiment, the proposed controller outperformed the admittance controller across all of RMSE, overshoot, and settling time. The admittance controller struggled to respond to sudden shocks because its coefficients were fixed. In contrast, as the proposed controller had learned the dynamic characteristics of the system, it could ensure more precise control performance under shocks. Due to its feedback-loop control characteristic, the admittance controller adjusted the output depending on the current force error, which led to a delay in tracking the target force. Hence, it exhibited a rapid overshoot during the experiments. In contrast, the proposed controller, by utilizing the currently measured force and the future target force trajectory as inputs, achieved stable control even during sudden force changes.

In the sinusoidal tracking experiment, the proposed controller again achieved lower RMSEs than the admittance controller for both the 0.5 Hz and 1 Hz forces. The admittance controller showed irregular peak values during the 0.5 Hz experiment. As mentioned earlier, the admittance controller’s slower tracking performance was due to its feedback-loop control. In contrast, the proposed controller could respond immediately to the input, thus achieving rapid tracking of the target value. In future work, we plan to extend the proposed controller to legged locomotion by implementing it on the legs of a bipedal robot.

## 5. Conclusions

This study proposed a DNN-based inverse model to address the limitations of admittance controllers for nonlinear systems. Furthermore, to eliminate the steady-state errors inherent in direct control with inverse models, we integrated a PI controller into our DNN-based inverse model to establish a learning-based contact force controller. To evaluate the performance of the proposed method, we designed a robotic leg and implemented the controllers on the developed platform to conduct comparative experiments. A step-response experiment on a robotic leg verified that augmenting the DNN-only inverse model with a PI controller successfully removed the steady-state error. The proposed controller was then evaluated against an admittance controller through step-response and sinusoidal-tracking experiments. The proposed controller outperformed the admittance controller, achieving a 66.3% average reduction in RMSE and, in step-response tests, 96% lower overshoot and 61% faster settling time on average.

## Figures and Tables

**Table 1:** Hardware specifications of robotic leg.

Part	Manufacturer/ Model Name	Key Specifications	
Joint 1	T-Motor (Nanchang, China)/AK10-9	Rated voltage	24–48 V
		Rated torque	18 Nm
		Rated current	10.6 A
Joint 2		Rated speed	228 rpm
		Gear ratio	9:1
		Motor weight	960 g
Joint 3	MyActuator (Suzhou, China)/RMD-X8	Rated voltage	24–48 V
		Rated torque	9 Nm
		Rated current	4.3 A
		Rated speed	170 rpm
		Gear ratio	9:1
		Motor weight	630 g
F/T sensor	Robotous (Seongnam, Republic of Korea)/RFT80-6A02	Rated Force	400 N
		Rated Torque	20 Nm
		Force Resolution	0.08 N
		Torque Resolution	0.004 Nm
		Overload capacity	300% FS
		Weight	294 g

**Table 2:** Training hyperparameters for first DNN-based inverse model.

Neural network structure	Input layer Hidden layer ① Layer 1: FC (Number of nodes: 16) ② Layer 2: FC (Number of nodes: 16) ③ Layer 3: FC (Number of nodes: 16) Output layer
Weight regularization	Hidden layer ① Layer 1: FC (L1: 1 × 10^3^, L2: 1 × 10^3^) ② Layer 2: FC (L1: 1 × 10^4^, L2: 1 × 10^3^) ③ Layer 3: FC (L1: 1 × 10^4^, L2: 1 × 10^3^)
Weight initialization	He normal initializer
Activation function	ReLU
Test/validation data	60%/20%
Learning rate	0.001
Epoch count	1000
Early stopping	500 epochs
Optimizer	Adam

**Table 3:** Results of 50 N step-reference experiments for 0.06-m-high obstacle.

Reference	50 N	
Controller	Admittance	DNN
RMSE (N)	8.46	2.39
Overshoot (%)	188.47	12.84
Settling time (s)	0.63	0.38

**Table 4:** Results of 50 N step-reference experiment for 0.06-m-high obstacle with and without PI controller.

Reference	50 N	
Controller	Without PI	With PI
RMSE (N)	2.39	1.43
Overshoot (%)	12.84	6.97
Settling time (s)	0.38	0.19

**Table 5:** Results of tracking performance for the admittance controller and the proposed controller (DNN + PI) in step-reference and sinusoidal experiments.

Experiment	Metric	Admittance	DNN + PI
Step, h = 0.06 m, ref = 50 N	RMSE (N)	8.46	1.43
	Overshoot (%)	188.47	6.97
	Settling time (s)	0.63	0.19
Step, h = 0.07 m, ref = 50 N	RMSE (N)	8.07	1.83
	Overshoot (%)	146.91	5.60
	Settling time (s)	0.76	0.36
Sine, h = 0.06 m, f = 0.5 Hz	RMSE (N)	5.81	3.38
Sine, h = 0.06 m, f = 1 Hz	RMSE (N)	10.78	4.01

## Data Availability

The data gathered during this study are available upon request from the first author.
